# Maternal complications in a geographically challenging and hard to reach district of Bangladesh: a qualitative study

**DOI:** 10.12688/f1000research.9445.1

**Published:** 2016-09-28

**Authors:** Animesh Biswas, Koustuv Dalal, Abu Sayeed Md Abdullah, Mervyn Gifford, MA Halim

**Affiliations:** 1 School of Health Sciences, Örebro University, Örebro, Sweden; 2 Centre for Injury Prevention and Research (CIPRB), Dhaka, Bangladesh

**Keywords:** Maternal complications, deaths, rural community, Bangladesh

## Abstract

**Background:** Maternal complications contribute to maternal deaths in developing countries. Bangladesh still has a high prevalence of maternal mortality, which is often preventable. There are some geographically challenging and hard to reach rural districts in Bangladesh and it is difficult to get information about maternal complications in these areas. In this study, we examined the community lay knowledge of possible pregnancy complications. We also examined the common practices associated with complications and we discuss the challenges for the community.

**Methods:** The study was conducted in Moulvibazar of north east Bangladesh, a geographically challenged, difficult to reach district. Qualitative methods were used to collect the information. Pregnant women, mothers who had recently delivered, their guardians and traditional birth attendants participated in focus group discussions. Additionally, in-depth interviews were conducted with the family members. Thematic analyses were performed.

**Results: **The study revealed that there is a lack of knowledge of maternal complications. In the majority of cases, the mothers did not receive proper treatment for maternal complications.

There are significant challenges that these rural societies need to address: problems of ignorance, traditional myths and family restrictions on seeking better treatment. Moreover, traditional birth attendants and village doctors also have an important role in assuring appropriate, effective and timely treatment.

**Conclusions:**  The rural community lacks adequate knowledge on maternal complications.  Reduction of the societal barriers including barriers within the family can improve overall practices. Moreover, dissemination of adequate information to the traditional birth attendant and village doctors may improve the overall situation, which would eventually help to reduce maternal deaths.

## Introduction

Maternal complications during pregnancy, delivery and after delivery contribute to deaths which are preventable in the majority of cases ^1, 2^. Every year, around 350,000 maternal deaths occur globally due to maternal complications, most of which are in developing countries ^3, 4^. Complications leading to maternal deaths can also lead to death of the baby if the mother dies during pregnancy. Bangladesh is facing some challenges to reduce maternal deaths. In Bangladesh there has been a reduction in maternal death during the last few decades and new goals have been set to meet the United Nations Sustainable Developmental Goal 3 by 2030 ^5^. Recent literature has clearly shown that the majority of the pregnant mothers in rural Bangladesh who died had suffered post-partum haemorrhage and eclampsia ^6^. Studies also showed that a lack of knowledge and literacy on maternal health issues including complications, health seeking behavior, and social and cultural beliefs also contribute to maternal deaths in rural Bangladesh ^3, 7– 11^. Similar studies in other developing countries have shown that the rural communities have a lack of knowledge about maternal complications ^9– 11^. Despite deliveries at specialist units have increased in the last few decades, home deliveries remain the most common, and are conducted by traditional birth attendants or family relatives ^12– 15^. Thus, risks of maternal complications are still high. It has also been observed that the involvement of village doctors and traditional birth attendants (TBA) during delivery might delay hospital referral during any maternal complications ^12– 19^.

 A recent study from Bangladesh showed that family and community are important players in reducing maternal and neonatal deaths ^6^. Therefore, the focus of the current study was to investigate the current knowledge, practices and challenges in the rural community. Information of such factors could be useful in designing interventions to reduce maternal deaths in Bangladesh.

## Methods

The Moulavibazar district of Bangladesh was chosen for the study. This district is one of the hard to reach districts of Bangladesh because of its geographical location. The district is surrounded by hills and a large number of tea plantations.

The community people residing surrounding the tea plantations were mostly from the local ethnic group. Three sub-districts (upazilas) were randomly chosen. Both focus group discussions (FGDs) and in-depth interviews (IDIs) were conducted ( Table 1).

**Table 1.** List of participants in the qualitative study.

Qualitative instruments	Age range	Participants
FGDs (n=5)	18 – 50 years	Pregnant mothers, mothers who recently delivered, female guardians, male guardians and traditional birth Attendants
IDIs (n=15)	25 – 60 years	Female guardians and male guardians of deceased mothers.

In Bangladesh, especially in the hard to reach areas parents, it is usual for mothers-in-law and fathers-in-law to be involved in making major decisions in families. Considering the objective and study context, five focus group discussions (FGDs) were conducted: one group of pregnant mothers, one group of recently delivered mothers, one group of mothers-in-law and mothers (of the pregnant/mothers who delivered), one group of husbands, fathers-in-law and fathers (of the pregnant/mothers who delivered) and one group of traditional birth attendants (TBA). A total of 42 participants participated in the FGDs, where each group consisted of between seven and nine participants . The total number of pregnant mothers and recently delivered mothers (during last three months before interview) having a live birth in the community was obtained from different sources including field level government health workers, community volunteers, traditional birth attendant and village doctors. The research officers discussed with them to confirm the list of mothers to obtain the desired participants for FGDs. The participants were initially approached by the researchers and briefly explained the study objectives. The participants who were interested in enrolling were given further details of the objectives of the study. Fathers-in-law, fathers, mothers-in-law and mothers were selected from the pregnant or mothers who had delivered. The traditional birth attendant (TBA) group participated in the FGDs in one upazila. The remaining FGDs were conducted in two of each upazilas.

For in-depth interviews, families of mothers who had died as a result of maternal complications were identified from government health records. In three upazilas of Moulvibazar district, 15 maternal deaths occurred between January and March 2014. Members of the families of the mothers who died were interviewed. We chose a family member who was present at the time of the maternal complications before the deaths, or who knew the mothers’ circumstances before the death. A total number of 15 IDIs were conducted at the 15 deceased households.

Two research officers with expertise in conducting qualitative research from an anthropological perspective conducted the study during May 2014. The research officers were provided training on how to conduct FGDs and IDIs in the community. Guidelines were developed, modified and pre-tested in the field before actual data collection. The guidelines are available as Supplementary material. During conduction of the FGDs, one of the research officers was responsible for facilitating the session, whilst another one was assigned to take key notes. All FGDs were conducted at the tea garden catchment area within the community.

For IDIs, face-to-face interviews were conducted with the respondents at the deceased’s homes. The research officer used guidelines to conduct the interviews. The objectives of the research were clearly mentioned to the respondents before data collection.

Each of the interviews was audio recorded and participants also gave informed consent for recording. Audio records were transcribed in the Bengali language and later translated into English. The accuracy was checked by translation and back-translation methods. Randomly selected transcripts were re-checked by the researchers and matched with the audio records and notes for its accuracy. Initially open coding of the data was performed and then selective code was used. A numbers of themes were identified after reading and re-reading the data ^20, 21^ and finally a thematic analysis was performed. Results were presented under three broad themes namely: perceptions, practices and challenges.

In Table 2 the content of the focus-group discussions and in-depth interviews are presented. Areas and prompts used during conduction were described here ( Table 2).

**Table 2.** Content of the focus-group discussion and in-depth interview.

Area of discussion	Types of prompts used
Perception on maternal complication in the community Practice during maternal complications in the community	Do you have any ideas on maternal complications? How and from whom did you come to know about these complications? If maternal complications are not known to you, why? What type of preparedness have you practiced in your family in case such complications arise? What are the practices in your community for seeking treatment? From which place and from whom you do treatment? If the community/you do not have a plan to prevent early complications, what are the reasons why?
Barriers for the family/ society/community	What are the social barriers in the community that make delay to seek better treatment? Are there any obstacles within the family that restrict mothers from managing maternal complications? Any suggestions/feedback to overcome the barriers?

Written informed consent to participate in the study (or a thumb print for consent of the illiterate) were taken from each of the respondents before the FGDs and interview sessions. The study received ethical permission from the Institutional Review Board of the Centre for Injury Prevention and Research, Bangladesh.

## Results

Ignorance, misperceptions and lack of knowledge followed by delivery conducted by local untrained birth attendants or family members at home increase risk of maternal death. Moreover, barriers in the society and traditional myth also influence the process. The findings from FGDs and IDIs are presented categorically under three sub-headings: perceptions, practices and challenges.

### Perceptions

Focus group discussions explored a higher level of perceptions on maternal complications among the pregnant mothers and the mothers who had recently delivered a live baby. This group also had some knowledge on what initiatives were required if any complications should arise. Female guardians of the families (mothers and mothers-in-law) had minimal knowledge of maternal complications, rather having a number of misperceptions. Male guardian groups (husbands, fathers and fathers-in-law) felt that the issues of maternal complications were not things men should be concerned about and that such issues were relevant only to the female members of the family.

In-depth interviews were performed with the family members of mothers who had died because of maternal complications. The majority of family members had no knowledge of maternal complications and there was a number of misconceptions. Many of the families felt that complications during pregnancy were common and they stated that they were resigned to such events as being in the hands of God.

One of the recently delivered mothers mentioned during the FGD

*“My legs were swollen and I had blurred vision in my last pregnancy. I knew these were complications of pregnancy and I went to the nearby government hospital with my husband for a health checkup*. *”*

A mother-in-law of a pregnant mother said during the FGD:

*“Vomiting, anorexia, some bleeding, fatigue, headaches, low or high blood pressure, loss of body weight, swelling of legs, and abdominal pain are usual during pregnancy, these are not complications. Only if there is a large amount of bleeding after delivery it is a complication for the mother”*.

One of the mothers of a deceased mother said in the IDI:

*“My daughter died due to our bad luck. Her face and legs were swollen during pregnancy. Such symptoms are common during pregnancy. We discussed it with our village doctor and the Dai (traditional birth attendant) and both of them ensured us not to be worried about this”*.

Pregnant mothers were asked how they obtained their information about maternal complications. During a FGD one of the pregnant mothers mentioned her source of knowledge:

*“I heard about maternal complications from a government health care provider during a courtyard meeting at our village. He talked about bleeding during pregnancy, swelling of legs and hands etc. I also learned such information on the television”*.

One of the husbands of a recently delivered mother mentioned in the FGD:

*“All the complications are naturally occurring during pregnancy and this is to do with female issues”*.

Another husband of a deceased mother said during the IDI:

*“As far as I know the mothers’ faces swell due to over feeding and taking too much rest during pregnancy”*.

### Practices

The majority of the responders in the female guardian and male guardian group mentioned during the FGD that they depended primarily on village doctors and traditional birth attendants for reducing complications such as swelling of the face and neck, and headaches and fevers. If major complications like bleeding or eclampsia occurred, then they went directly to the upazila or district hospital. Almost all the traditional birth attendants said they worked with the pregnant women without having received any training.

The majority of the female guardians (mothers and mothers-in-law) and male guardians (husbands and fathers-in-law) of the deceased mothers mentioned that when any complications in pregnancy occurred they depended solely on Kabiraj (quacks) and village doctors and traditional birth attendants for treatment. Some of them said they relied on the drug salesmen in the pharmacies. None of them said they had sought help from government or private agencies.

One of the mothers-in-law of a mother who had recently delivered mentioned during the FGD:

*“I provided a small amount of food and let my daughter-in-law to rest as her face and legs were swollen during her last pregnancy”*.

One of the husbands of a mother who had recently delivered said:

*“My wife’s legs and face were swollen and she suffered from blurred vision and headaches during her last pregnancy. When she had not recovered with the village doctor’s treatment, I then took her to Upazila Government hospital and she was cured with treatment*. *”*

During the FGD one of the TBA mentioned:

*“I conducted more than a hundred deliveries in the community. Due to cessation of menstruation during pregnancy heavy bleeding can occur after delivery. I can handle any difficult complications such as obstructed and prolonged labor without any instruments. I learn these traditionally without any training”*.

 During an IDI one mother of a deceased mother said:

*“My daughter had high fever along with severe bleeding after delivery. The village Kabiraj (quack) provided her with herbal drugs but when my daughter didn’t get better with traditional treatments I contacted the village doctor and he gave some drugs. But unfortunately my daughter died with a high fever seven days after delivery”*

During an IDI one of the husbands said:

*“I heard from our village doctor that my wife’s face was swollen due to decreased urination and working less during pregnancy. I then advised her to work more but it is our bad luck that she died immediately after delivery at home”*.

### Challenges

Fifty percent of the male respondents in FGDs and the majority of males participated in IDIs had no knowledge about maternal complications. During discussions it emerged that males had a misperception on this issue and thought that complications are a normal phenomenon during pregnancy. In FGDs, about 90% of mothers who had recently delivered and pregnant women mentioned that the main source of problems during maternal complications are the female decision makers in the family including the mother and mother-in-law. Most of the participants in the IDIs mentioned that they depended on village doctors and traditional birth attendants for advice and treatment for complications. They also thought that treatment of maternal complications is expensive at government facilities. TBAs and village doctors are not very aware of maternal complications and consider complications during pregnancy normal. They still practice some traditional procedures based on myths. Almost all of the participants stated that information about maternal complications should be made available to the head of the family. They recommended raising community awareness of maternal complications by use of through posters, videos, health camps and courtyard meetings. They also suggested more involvement of government health workers in the community to help inform older women in the family on maternal complication. The majority of the male groups in FGDs mentioned that they prefer home delivery by traditional birth attendants to the check up and deliveries conducted by male doctors in hospital.

A mother who had recently delivered said during a FGD:

*“I had severe headaches during my pregnancy. My legs were swollen. I wanted to go to hospital but my husband and mother-in-law didn’t agree as they thought these symptoms were normal during pregnancy”*.

One of the TBAs in a FGD stated:

*“I have conducted deliveries for many years. Swelling of legs, blurred vision, headaches, anemia and vomiting are normal signs during pregnancy. I even delivered a mother after a labor of three days”*.

 One of the husbands of a deceased mother said during an IDI:

*“When the traditional birth attendant could not advance my wife’s delivery I called the village doctor and he injected a drug to facilitate delivery but then after delivery she started to bleed and then the village doctor arrived and injected saline. One of my neighbors advised me to take my wife to hospital. But I have no enough money for hospital treatment. She died the day after delivery”*.

## Discussion

Our study showed that mothers who had recently delivered and the pregnant women group have good knowledge on maternal complications but cannot put this in practice due to social and family barriers in their local communities. Both male and female guardians of mothers who recently delivered have more awareness of maternal complications and practices than the guardians of deceased mothers. Male guardians have less knowledge of practice during maternal complications. However, the mother and mother-in-law of deceased mothers traditionally believed that complications are normal during pregnancy.

Mothers who had recently delivered were more aware of maternal complications and conducted health checkup by community health care providers during any complication compared to the families of deceased mothers, indicating that health care during pregnancy may reduce the chance of maternal mortality ^17^.

Our study explored that the male guardians in the family such as husbands, fathers and fathers-in-law were not aware of maternal care during pregnancy. They were ignorant of maternal complication issues. Literature indicated that husbands are important for improving maternal health care and maternal complications ^11^. Therefore, husbands could be encouraged to insist that their wives use health care facilities, especially during any maternal complications. Husbands also could provide financial support to their wives and encourage adequate nutrition.

There were many misperceptions and malpractices on maternal complications in the communities, which supported the finding of a study describing the prevalence of reported complications by analyzing the risk factors of obstetric complications in rural setting of Bangladesh ^22^.

Many pregnant mothers and mothers who had recently delivered had good awareness of maternal complications, but could not practice due to family barriers. Husbands' social and economic supports could eliminate or reduce barriers like high costs, poor transportation, and long distances to health care facilities. They could also facilitate other factors associated with for improved utilization of delivery care ^23^.

The information provided by all participants in the survey underlines the importance of mass awareness among community people, which, if improved, can eliminate misperception and malpractices during maternal complications. Appropriate health care seeking behavior among community people will be a first step to improve maternal health services and ultimately reduce maternal death ^24^.

## Conclusion

Community perception on maternal complications was inadequate among the male and female guardian group of mothers who recently delivered and the group who had maternal deaths in their family. There was scarcity of knowledge among male respondents in both groups; therefore, decision making during maternal complications was missing in the majority of cases. Mothers and mothers-in-law in a family also required knowledge improvements to develop their practice. We observed that communities are still dependent on traditional birth attendants to deliver babies in most of the cases. Focused interventions are required by the government at the local level to improve the overall situation of maternal complications which will consequently reduce maternal deaths in Bangladesh.

## Data availability

The data referenced by this article are under copyright with the following copyright statement: Copyright: © 2016 Biswas A et al.

Raw datasets have not been made available at the request of the ethics committee in order to maintain participant confidentiality. This data is stored at the Department of Reproductive and Child Health Unit of CIPRB, and is available upon request. Please contact the corresponding author for further information.

## Supplementary material

English translation of the guidelines used for the qualitative study of maternal complications in a geographically challenging and hard to reach district of Bangladesh.

## References

[1] Maternal or pregnancy comlication [Internet]. Reference Source

[2] Thompson JF Roberts CL Currie M: Prevalence and persistence of health problems after childbirth: Associations with parity and method of birth. *Birth.* 2002;29(2):83–94. 10.1046/j.1523-536X.2002.00167.x 12051189

[3] World Health Organization (WHO): Trends in Maternal Mortality: 1990 to 2008. Estimates developed by WHO, UNICEF, UNFPA and The World Bank.2010 Reference Source

[4] Zahr CA Tessa W: Antenatal care in developing countries: promises, achievements and missed opportunities: an analysis of trends, levels and differentials. World Health Organ.2003 Reference Source

[5] Sustainable Development Goals. Reference Source

[6] Biswas A: Maternal and Neonatal Death Review System to Improve Maternal and Neonatal Health Care Services in Bangladesh. Örebro University, Sweden,2015 Reference Source

[7] GBD 2013 Mortality and Causes of Death Collaborators. Global, regional, and national age-sex specific all-cause and cause-specific mortality for 240 causes of death, 1990–2013: a systematic analysis for the Global Burden of Disease Study 2013. *Lancet.* 2015;385(9963):117–71. 10.1016/S0140-6736(14)61682-2 25530442 PMC4340604

[8] Karkee R Baral OB Khanal V: The role of obstetric knowledge in utilization of delivery service in Nepal. *Health Educ Res.*England;2014;29(6):1041–8. 10.1093/her/cyu059 25274718

[9] Iyengar K Yadav R Sen S: Consequences of maternal complications in women's lives in the first postpartum year: a prospective cohort study. *J Health Popul Nutr.* 2012;30(2):226–40. 10.3329/jhpn.v30i2.11318 22838164 PMC3397333

[10] Khan AR Jahan FA Begum SF: Maternal mortality in rural Bangladesh: the Jamalpur District. *Stud Fam Plann.*United States;1986;17(1):7–12. 10.2307/1966950 3485842

[11] Ahmed A Hossain SAS Quaiyum A: Husbands’ knowledge on maternal health care in rural Bangladesh: An untapped resource? *Trop Med Int Heal.* 2011;16:291.

[12] BBS.2001 Reference Source

[13] BBS.2010 Reference Source

[14] Shabnam J Gifford M Dalal K: Socioeconomic Inequalities in the use of delivery care services in Bangladesh: A comparative study between 2004 and 2007. *Health.* 2011;3(12):762–71. 10.4236/health.2011.312127

[15] Andrews JY Dalal K: Umbilical cord-cutting practices and place of delivery in Bangladesh. *Int J Gynaecol Obstet.* 2011;114(1):43–6. 10.1016/j.ijgo.2011.01.025 21571269

[16] Ronsmans C Graham WJ Lancet Maternal Survival Series steering group: Maternal mortality: who, when, where, and why. *Lancet.* 2006;368(9542):1189–200. 10.1016/S0140-6736(06)69380-X 17011946

[17] Moran AC Winch PJ Sultana N: Patterns of maternal care seeking behaviours in rural Bangladesh. *Trop Med Int Heal.* 2007;12(7):823–32. 10.1111/j.1365-3156.2007.01852.x 17596248

[18] Markos D Bogale D: Birth preparedness and complication readiness among women of child bearing age group in Goba woreda, Oromia region, Ethiopia. *BMC Pregnancy Childbirth.*England;2014;14:282. 10.1186/1471-2393-14-282 25132227 PMC4148918

[19] Moghani Lankarani M Changizi N Rasouli M: Prevention of pregnancy complications in iran following implementing a national educational program. *J Family Reprod Health.*Iran;2014;8(3):97–100. 25628717 PMC4275557

[20] Irving S: Interviewing as Qualitative Research - A Guide for Researchers in Education and the Social Sciences.Teach Coll Columbia Univ USA.2006 Reference Source

[21] Boyatzis RE: Transforming qualitative information: Thematic analysis and code development. Sage.1998 Reference Source

[22] Sikder SS Labrique AB Shamim AA: Risk factors for reported obstetric complications and near misses in rural northwest Bangladesh: analysis from a prospective cohort study. *BMC Pregnancy Childbirth.*England;2014;14:347. 10.1186/1471-2393-14-347 25282340 PMC4287506

[23] Story WT Burgard SA Lori JR: Husbands’ involvement in delivery care utilization in rural Bangladesh: A qualitative study. *BMC Pregnancy Childbirth.* 2012;12:28. 10.1186/1471-2393-12-28 22494576 PMC3364886

[24] Quayyum Z Khan MN Quayyum T: “Can community level interventions have an impact on equity and utilization of maternal health care” - evidence from rural Bangladesh. *Int J Equity Health.* 2013;12:22. 10.1186/1475-9276-12-22 23547900 PMC3620556

