# Supplementary material for: Maternal complications in a geographically challenging and hard to reach district of Bangladesh: a qualitative study
Source: F1000Res. 2016 Sep 28;5:2417. [Version 1] doi: 10.12688/f1000research.9445.1 (PMC5089125; doi:10.12688/f1000research.9445.1)
Supplement: Supplementary file 1 [file f1000research-5-10173-s0000.tgz › 7d87eb97-fb21-4f2d-b640-314ee924970d.doc]

**Maternal complications in a geographically challenging and hard to reach district of Bangladesh: a qualitative study**

**Guideline for qualitative study**

**Focus Group Discussion**

(Target Population: Pregnant mothers, mothers who recently delivered, female guardians, male guardians and traditional birth attendants)

**Perception of maternal complication in the community**

- Do you have any ideas on maternal complications?
- What types of complications usually occurred during pregnancy, delivery & after delivery period.?
- How and from whom did you come to know about these complications?
- If maternal complications are not known to you, why do you think such maternal complications occur?

**Practices during maternal complication in the community**

- What type of preparedness have you practiced in your family in case such complications arise?
- What is the practice in your community for seeking treatment? From which place and from whom?
- If the community / you do not have a plan to prevent early complications, what are the reasons why?

**Barriers for the family/ society/ community**

- What are the social barriers in the community that delay persons to seek better treatment?
- Are there any obstacles within the family that restrict mothers from managing maternal complications?

**In-depth Interview**

(Target Population: Female and male guardians of deceased mothers)

**Perception on maternal complication**

- What are your perceptions of maternal complications?
- How and from whom did you come to know about these complications?
- If maternal complications are not known to you, why do you think such maternal complications occur?

**Practice on maternal complications**

- What actions did you/ your family members or community people take during the maternal complications of the deceased mother?
- Did you/ other family member seek treatment or management for the mother?
- Where and to whom did you go for treatment of the deceased mother?

**Barriers at the family/ society/ community**

- Are there any barriers in the family/ society /community to seek treatment/ management during maternal complications?
- Are there any obstacles within the family that restrict mothers from managing maternal complications?
- Do you have any suggestions/ feedback to help overcome societal barriers to treating/managing maternal complications?
