# Supplementary material for: Maternal complications in a geographically challenging and hard to reach district of Bangladesh: a qualitative study
Source: F1000Res. 2016 Sep 28;5:2417. [Version 1] doi: 10.12688/f1000research.9445.1 (PMC5089125; doi:10.12688/f1000research.9445.1)
Supplement: Peer review history

# Peer review: Referee response for version 1

**DOI:** 10.5256/f1000research.10173.r17019

**Published:** 2016-10-17

**Authors:** Asish Kumar Das

**Affiliations:** 1 Médecins Sans Frontières International , Dhaka, Bangladesh

**Title and Abstract**: The abstract is well written. The title of the study perhaps would have been a little more expressive if it would have a word “Rural”. The study has been conducted in the rural areas and there are differences in perception among the rural and urban population in terms of knowledge, perception and practice.

**Article Content:** It is a well-designed study. The study population groups have been selected appropriately, FGD groups were homogenous in nature which was crucial in order to get the best out of a FGD. The discussions were well directed. Analysis was done manually and no qualitative software has been used which is OK (and perhaps more meaningful). The study looked into the issue of maternal deaths from all possible perspectives. As a public health professional with program based hands on experience in maternal and child health, I have repeatedly come across the issues/ difficulties as mentioned in the article. I consider it a real good one.

**Conclusion**: The conclusion was comprehensive and was able to summarize the study efficiently.

**Data:** As it is a qualitative study, I was not expecting any set of data. The quotes from the respondents were enough for me.  

I have read this submission. I believe that I have an appropriate level of expertise to confirm that it is of an acceptable scientific standard.

# Peer review: Referee response for version 1

**DOI:** 10.5256/f1000research.10173.r16673

**Published:** 2016-10-10

**Authors:** Puspa Raj Pant

**Affiliations:** 1 Centre for Child and Adolescent Health, University of the West of England, Bristol, UK

I would like to congratulate the authors for their efforts to compile such evidences required for the improvement of maternal and child health service utilisation in Bangladesh as well similar communities in neighbouring countries. 

 I find the manuscript well structured and written. However, I suggest some editing for languages (please see highlighted and suggested in the pdf text) for consistency and rearrange the quotations in the result section.

 I have also suggested to touch the situation of Antenatal Care service use by the women in the study area and briefly describe the existing government/nongovernmental structures available in the communities.

 I approve the manuscript to be indexed after the incorporation of the comments and suggestions.

I have read this submission. I believe that I have an appropriate level of expertise to confirm that it is of an acceptable scientific standard.
